# Prediction of difficult laparoscopic cholecystectomy: An observational study

**DOI:** 10.1016/j.amsu.2021.103060

**Published:** 2021-11-14

**Authors:** Tika Ram Bhandari, Sarfaraz Alam Khan, Jiuneshwar Lal Jha

**Affiliations:** a Department of General Surgery, People's Dental College and Hospital, Kathmandu, Nepal

**Keywords:** Cholelithiasis, Predictors, Difficult laparoscopic cholecystectomy

## Abstract

**Background:**

Laparoscopic cholecystectomy (LC) is a gold standard treatment of symptomatic gallstone disease. Meanwhile, it is also a challenging procedure demanding excellent expertise for the best outcomes. Many times, difficult laparoscopic cholecystectomy is a nerve-wracking situation for surgeons. It endangers patients by causing potential injury to vital structures. Thus, we aimed to identify predictors for difficult LC.

**Methods:**

A retrospective cross-sectional review of surgical records was done. Patients who underwent laparoscopic cholecystectomy on an elective basis from July 2017 to June 2021 were included in the study. We divided our patients into two groups based on operative findings of difficult LC; difficult LC group and non-difficult LC group. We compared patient's demographics, predictors, and perioperative details and analyzed the data.

**Results:**

A total of 338 patients (82 males) with a median age of 47 years were studied. Total difficult LC was found in 52 patients (15.4%). The overall conversion rate was 8.9%. Logistic multivariable regression analysis revealed that; male gender (odds ratio (OR); 0.171, confidence interval (CI),(0.043–0.675), P; 0.012), past history of acute cholecystitis (OR; 0.038, CI; (0.005–0.309), P; 0.002), gall bladder wall thickness (≥4–5 mm) (OR; 0.074, CI; (0.008–0.666), P; 0.020), fibrotic gallbladder (OR; 166.6, CI; (7.946–3492), P; 0.001), and adhesion at Calot's triangle (OR; 0.021, CI (0.001–0.311), P; 0.005) were independent predictors of difficult LC.

**Conclusions:**

Gender (male), past history of acute cholecystitis, gallbladder wall thickness (≥4–5 mm), fibrotic gallbladder, and adhesion at Calot's triangle are significant predictors for difficult LC. Moreover, an awareness about reliable predictors for difficult LC would be helpful for an appropriate treatment plan and application of the resources to anticipate difficult LC.

## Highlights

• Cholecystolithiasis is one of the common causes of hospital admission throughout the world.
• Laparoscopic cholecystectomy (LC) is a gold standard treatment of symptomatic gallstone disease.
• Many times, difficult laparoscopic cholecystectomy is a nerve-wracking situation for surgeons which affects patient.
• An awareness of reliable predictors for difficult LC would be helpful for an appropriate treatment plan.

## 1 Introduction

Laparoscopic cholecystectomy (LC) is the gold standard treatment for symptomatic cholelithiasis due to its effectiveness, and safety. Moreover, the benefits of laparoscopic cholecystectomy are; less postoperative pain, faster recovery, earlier return of bowel function, and shorter hospital stay when compared to conventional cholecystectomy [1,2]. Although, the LC is the most common operation performed these days, some of the intended LC require conversion due to several factors. Many a time it demands conversion to open cholecystectomy due to intraoperative complications for the safe ending of the procedure and takes more than anticipated time. However, current literature has mentioned a conversion rate of nearly about (2%–10%) [3].

Most of the time, the levels of difficulties are hard to assume. Nevertheless, it is necessary to predict so that the patient can be informed regarding the possibility of conversion. Likewise, the surgeon can get mentally prepared to be ready for having a good surgical team, intraoperative cholangiogram, the timing of the procedure and have overall preparedness to defy the difficult cholecystectomy for better postoperative outcomes. It is always better to know the predictors to complete such challenging surgeries. Based upon the risk factors like; patient demographics such as age, gender, body weight, comorbidity, and ASA score, along with clinical findings (acute versus chronic cholecystitis), and the surgeon's experience; the chance of possible complications, and conversion into open surgery can be estimated [4,5].

Therefore, the knowledge about consistent predictors for difficult cholecystectomy would be particularly beneficial to not only to set appropriate management plan but also to assemble available resources to defy difficult LC. Thus, this study aimed to determine predictors for difficult LC.

## 2 Methods and methods

A retrospective cross-sectional review of surgical records was done for all patients who underwent laparoscopic cholecystectomy on an elective basis at the People's Dental College and Hospital, Teaching hospital, Kathmandu, Nepal from July 2017 to June 2021. The study was approved by the Institutional Review Committee of People's Dental College and Hospital, Kathmandu, Nepal. We included only those patients who were planned for elective LC and excluded those who underwent emergency LC for cholelithiasis with acute cholecystitis. We divided the patients into two groups based on operative findings of difficult LC; difficult LC group and non-difficult group. We defined difficult LC as those comprising an operative time of more than two hours, need for conversion to open cholecystectomy, significant bleeding (any hemorrhage that couldn't be managed with manual pressure and cautery and had to be managed with conversion into open surgeries) and those with vascular and biliary injuries.

Patient's demographics, risk factors, and perioperative details were analyzed. Clinical evaluation, abdominal ultrasonography as well baseline investigations were used as a tool to assess all patients for surgery. The patients who presented with acute phase (acute cholecystisis) was planned for surgery after the resolution of symptoms. Therefore, all of these patients were initially managed medically, discharged and readmitted for elective laparoscopic cholecystectomy.

Meanwhile, the selected patients with suspected choledocholithiasis or with dilated biliary duct in ultrasonography had been evaluated with magnetic resonance cholangiopancreatography (MRCP). In addition, patients with common bile duct stones had endoscopic retrograde cholangiopancreatography (ERCP) before cholecystectomy.

During operation, all patients received perioperative prophylactic antibiotics. The standard four-port technique was applied for the procedure. Meanwhile, the open method was used to introduce a sub umbilical cannula. In most of the cases, we placed titanium clips for cystic artery and cystic duct ligations and we did not perform intraoperative cholangiography routinely. Furthermore, we only put a closed suction drain in selected cases according to need.

Perioperative data comprising duration of surgery, and operative findings were documented. During analysis of data, independent *t*-test or Mann–Whitney test was applied for all quantitative variables while Chi-square test or Fisher's exact test was used for all categorical variables. Univariate and multivariate logistic regression were performed to recognize independent predictors associated with difficult LC. Statistical software SPSS version 25.0 (Statistical Package for the Social Sciences) was used for statistical analysis. A P value < 0.005 was considered statistically significant.

Our work is fully compliant with the STROCSS criteria www.strocssguideline.com in which a completed STROCSS checklist stating the page numbers [6].

## 3 Results

A total of 338 patients who underwent laparoscopic cholecystectomy (52 patients with difficult and 286 patients without difficult LC) were studied during the study period. The majority of patients 256 (75.7%) were female with a female to male ratio of 3:1. The median age was 47 years. Patients' demographics and perioperative data are shown in Table 1. When we assessed different preoperative and intraoperative variables in two groups (difficult LC and non-difficult LC), we found differences based upon age, gender, past history of acute cholecystitis, gallbladder wall thickness, fibrotic gallbladder, and adhesion at the triangle of Calot (*P* < 0.05), Table 1. However, We did not observe significant differences in other variables (P > 0.005).

**Table 1:** Patient's demographics and perioperative data.

Variable	Difficult LC		P value
	N = 52 (%)	Non- Difficult LC N = 286 (%)	
**Age, (years)**			0.019*
**≥60**	12(23.1)	32 (11.2)	
**<60**	40 (76.9)	254(88.8)	
**Sex**			0.001*
**Male**	36 (69.2)	46 (16.1)	
**female**	16 (30.8)	240(83.9)	
**Smoking**			0.275
**Smoker**	12 (23.1)	48(16.8)	
**Non-smoker**	40 (76.9)	238(83.2)	
**Past history of acute** **cholecystitis**			0.001*
**Yes**	42 (80.8)	11 (3.8)	
**No**	10 (19.2)	275 (96.2)	
**BMI**^**♣**^			0.516
**<18.5**	2(3.8)	9 (3.1)	
**≥18.5 < 25**	47 (90.4)	269 (94.1)	
**≥25**	3 (5.8)	8 (2.8)	
**Comorbidity,%**	10 (16.1)	28 (8.4)	0.012
**Diabetes mellitus**	7 (13.5)	7(2.4)	
**Hypertension**	3 (5.8)	9 (2.9)	
**Cardiovascular disease**	0 (0)	4 (1.4)	
**Respiratory diseases**	0 (0)	2 (0.7)	
**Neurological problems**	0 (0)	2 (0.7)	
**Renal disease**	0 (0)	4 (1.4)	
**GB**^**♦**^**wall thickness (mm)**			0.001*
**≥4**–**5**	46(88.5)	11(3.8)	
**<4**–**5**	6 (11.5)	275 (96.2)	
**Fibrotic GB**^**♦**^			0.005*
**Yes**	12(23.1)	27(9.4)	
**No**	40(76.9)	259 (90.6)	
**Adhesion in TOC**^**†**^			0.001*
**Yes**	44(84.6)	7(2.4)	
**No**	8 (15.4)	279 (97.6)	

Continuous variables are presented as median, Categorical variables are presented as n (%); ^♣^BMI; Body mass index, ^¶^ASA; American society of anesthesiology, ^†^TOC; Triangle of Calot; ^♦^GB; Gallbladder, *P; value is significant if < 0.05.

We also assessed the variables defining difficult laparoscopic cholecystectomy and we observed conversion to open surgery in 30 (8.9%) patients; operative time (≥120 mins) in 13 (3.8%) patients and significant hemorrhage requiring manual or synthetic hemostasis in 9 (2.7%) patients. However, we did not observe any injury to the bile duct or bowel. Failure to adequately visualize the biliary tract anatomy and Calot's triangle due to intense fibrosis was the most common cause of conversion in our series, followed by intense intraoperative bleeding. However, most of the time the primary hemorrhage was due to injury to the cystic artery during the dissection within the frozen field around the triangle of calot's that was managed with the manual or synthetic application of hemostases.

We had also evaluated different factors associated with difficult LC. In logistic regression analysis, on multivariate analysis gender (male), past history of acute cholecystitis, gallbladder wall thickness (≥4–5 mm), fibrotic gallbladder, and adhesion at the triangle of Calot were significantly associated with an increased risk of difficult laparoscopic cholecystectomy (P < 0.05) which is shown in Table 2.

**Table 2:** Association of variables with difficult laparoscopic cholecystectomy.

Variables	Difficult LC n = 52 (%)	Univariate analysis OR,(CI) P value	Multivariate analysis OR;(CI); P* value
Age(≥60)	12 (23.1)	2.06; (1.133–5.004); 0.019*	0.231;(0.044–1.205); 0.082
Sex (Male)	36 (69.2)	4.30; (3.12–5.93); 0.001*	0.171;(0.043–0.675); 0.012*
Presence of Comorbidity	10 (19.2)	1.96; (1.01–3.79); 0.047*	0.729;(0.120–4.429); 0.731
Past history of acute cholecystitis	42 (80.8)	21.0; (11.58–38.05); 0.001*	0.038; (0.005–0.309); 0.002*
GB^♦^ wall thickness ≥4–5 mm	46(88.5)	23.0; (12.77–41.39); 0.001*	0.074; (0.008–0.666); 0.020*
Fibrotic GB^♦^	12(23.1)	2.44; (1.325–4.51); 0.005*	166.6; (7.946–3492); 0.001*
Presence of Adhesion in TOC^†^	44(84.6)	34.5; (16.48–72.51); 0.001*	0.021;(0.001–0.311); 0.005*

OR: odds ratio; CI: confidence interval; ^♦^GB; Gallbladder; ^†^TOC; Triangle of Calot; *P; value is significant if < 0.05.

## 4 Discussion

Over the past few decades, the benefit of laparoscopic cholecystectomy over open surgery has been extensively accepted. However, many times it is challenging and the surgeon has to face the difficulty that might lead to injury to adjacent structures leading to an increase in morbidity. Therefore, the preoperative estimate of a difficult LC is essential to predict the difficulty as well as for a better surgical plan. It also helps the surgeon in being better prepared to anticipate the intra-operative difficulties [7].

The literature has mentioned different predictors for difficult LC such as age 60 or more, male gender, comorbid condition, past history of acute cholecystitis, previous abdominal surgery, gall bladder wall thickness ≥4–5 mm, contracted gall bladder, and impacted stone [8,9].

When we analyzed the predictors of difficult LC in DLC, we found that gender (male), past history of acute cholecystitis, gall bladder wall thickness (≥4–5 mm), fibrotic gallbladder, and adhesion in the triangle of Calot were significant risk factors for difficult LC likewise reported in other studies [10].

In Our study we found that there was delayed presentation of symptoms by male as compared to female patients. The possible reason could be less attention to mild symptoms leading to presentation only after disease progression. This scenario has also been mentioned in other studies [[11], [12]].

Likewise, the elderly population (age >60 years) has been defined as a predictor for difficult laparoscopic cholecystectomy in some studies [11]. In our study, age, ASA, smoking status, BMI (obesity), presence of comorbidity have not been found as risk factors similar to other studies [13].

Notably, Rassan et al. in their study reported that BMI (obesity) is an important predictor [5]. But in our study, we could not assess since none of our patients were obese instead we found some malnourished patients in our study but that did not have any effect on determining the difficult LC. Regarding surgeon's experience, in our institution, LC is regularly performed by consultant surgeons, so we did not include the experience of the surgeon as a predictor. However, Some studies maintained the operative inexperience of surgeons as a risk factor for difficult LC [14].

Patients who required hospitalization for repeated attacks of acute cholecystitis carry more chances of difficult laparoscopic cholecystectomy and conversion. Possibilities are dense adhesions at Calot's triangle and gall bladder fossa(9). In our study also, it was found to be a significant factor for the prediction of difficult laparoscopic cholecystectomy. These cases required more time for dissection of Calot's triangle and dissection of the gall bladder from the liver bed.

Moreover, we found fibrosis of the gallbladder is associated with difficult LC similar to reported by Stanisic et al. [10]. The fibrotic gallbladder usually resulted from repeated episodes of attack of cholecystitis due to constant irritation of the gallbladder wall with gallstones. Chronic inflammation of gall bladder leads to pericholecystic adhesion and adhesions at the triangle of calot’ that leads to difficulty in dissection during LC and this increased duration of surgery, increase the risk of bleeding and injuries to adjacent structures. So, adhesion at Calot's triangle is another important predictor described in a few studies [15,16] similar to our study.

In literature, the role of CT scan has been maintained in difficult LC [17]. However, we could not perform CT scans routinely because of the economic constraints of our patients. We perform the preoperative ultrasonographic evaluation of patients scheduled for surgery. Preoperative ultrasonographic findings of gallbladder wall thickness are also a significant predictor for difficult LC in our study, similarly reported by Giuseppe et al. [7]. Some studies have highlighted the use of laparoscopic ultrasound during cholecystectomy and incorporate its benefit in difficult situations while the anatomy is not clear [18].

To manage the challenging situations of difficult laparoscopic cholecystectomy, many studies have recommended alternative procedure and advised to follow a safe cholecystectomy principle [19]. Inconsistent with that, Gupta et al. in their study outlined the concept of culture of safety in cholecystectomy. They emphasized that this universal culture of safety should be routinely adopted by the whole surgical team during an individual case. The main components of this are concluded in following points: (a) a clear understanding of relevant anatomy; (b) appropriate and timely use of bailout techniques; (c) obtaining CVS before the division of cystic duct and artery in every case; (d) recognizing the importance of time-out; (e) use of intraoperative imaging; (f) obtaining a second opinion in difficult cases; and (g) importance of proper documentation [9].

Besides, several approaches have been described for the management of difficult LC in the literature including laparoscopic subtotal cholecystectomy, fundus first or antegrade or other techniques [20,21]. Though we usually performed laparoscopic subtotal cholecystectomy being very loyal to conversion, this study could not make a single recommendation about these techniques to manage difficult LC sand this has been agreed about growing consensus in laparoscopic subtotal cholecystectomy and fundus first methods.

We believe that this is one of the precise series clearly showing an association of different preoperative and intraoperative predictors with difficult LC. Regarding the limitations of our study, we acknowledge that this is a retrospective and single-centered study. Hence, to endorse our findings, we recommend conducting appropriately designed prospective studies in our setting in the future.

## 5 Conclusions

Gender (male), past history of acute cholecystitis, gallbladder wall thickness (≥4–5 mm), fibrotic gallbladder, and adhesion at Calot's triangle are significant predictors for difficult LC. Moreover, an awareness about reliable predictors for difficult LC would be helpful for an appropriate treatment plan and application of the resources to anticipate difficult LC.

## Ethical approval

The study was approved by the Institutional Review Committee of People's Dental College and Hospital, Kathmandu, Nepal.

## Funding

There are no sponsors involved in the study.

## Author contribution

All authors equally contributed to the study concept or design, data searching, data analysis or interpretation, writing the paper.

## Consent

Is a retrospective study. So Not applicable.

## Registration of research studies

1. Name of the registry: http://www.researchregistry.com
2. Unique Identifying number or registration ID: 6943
3. Hyperlink to your specific registration: https://www.researchregistry.com/browse-the-registry#home/

## Guarantor

Dr. Tika Ram Bhandari.

## Provenance and peer review

Not commissioned, externally peer-reviewed.

## Declaration of competing interest

All authors declare that they have no competing interests.
